# Modes of Committing Suicide in France

**Published:** 1849-10

**Authors:** 


*Modes of committing Suicide in France.* By M. Brierre de Boismont.

M. Brierre de Boismont furnishes the following table as the result of an examination which he has made of the returns of 4,595 suicides committed in the Department of the Seine. Death occurred in

| | |
|---|---|
| 1426 | by charcoal fumes, |
| 989 | by drowning, |
| 796 | by hanging, |
| 578 | by fire-arms, |
| 207 | by cutting instruments, |
| 158 | by poisoning, |
| 424 | by precipitation, |
| 16 | by being run over, |
| 1 | by abstinence. |
| 4595 | |

*Annales d' Hygiene*, tom. xi, p. 412.

